# Protective effects of lupeol on pesticides induced testicular and oxidative damage of male rats

**DOI:** 10.1007/s10735-025-10425-3

**Published:** 2025-05-08

**Authors:** Pınar Koroglu, Ismet Burcu Turkyılmaz Mutlu, Huseyin Us, Melis Coremen, Ayca Sezen Us, Omur Karabulut Bulan, Refiye Yanardag

**Affiliations:** 1 Department of Histology and Embryology, Faculty of Medicine, Haliç University, Istanbul, Turkey; 2 Department of Chemistry, Faculty of Engineering, Istanbul University-Cerrahpaşa, Istanbul, Turkey; 3 Department of Biology, Faculty of Science, Istanbul University, Vezneciler, Istanbul, Turkey

**Keywords:** Lupeol, Oxidative stress, Pesticide, Sperm smear, Testes

## Abstract

Pesticides are used as insecticides in agriculture. Lupeol (LUP) is a dietary triterpene with antioxidant effects. This experiment aimed to investigate the effects of LUP against testicular pesticides toxicity via histological and biochemical findings. Wistar albino rats were used. Control, corn oil, malathion (MAL), chlorpyrifos (CPF), tebuconazole (TEB), LUP, MAL + LUP, CPF + LUP, and TEB + LUP group. Control group rats were given physiological saline. Corn oil is used as a solvent in the preparation of pesticide agents. MAL 50 mg/kg, CPF 10 mg/kg, TEB 50 mg/kg (first 4 days) and 25 mg/kg (last 4 days), LUP 20 mg/kg were given via oral gavage. After 10 days, rats were dissected, and testes were taken for histological analysis. Oxidative stress parameters were determined spectrophotometrically in testicular tissue specimens. In the pesticide group, histological and biochemical damage score increased, morphological sperm smear defects were also detected. These defects were reversed upon the administration of LUP. LUP demonstrated an ameliorative effect on histopathological and biochemical parameters in pesticide induced testicular damage.

## Introduction

Acute and chronic exposure to pesticides have deleterious effects on all tissues and metabolism (Karami-Mohajeri et al. 2017).

Organophosphates containing MAL, CPF, parathion, diazinon and fenthion are a group of commonly used insecticidal pesticides (Suratman et al. 2015).

Pesticides exposed because of environmental or occupational exposure can directly affect semen and sperm parameters. The male endocrine system and hormones regulate the spermatogenic process. Pesticide metabolites can mimic male hormones or cause damage in testes tissue (Martenies and Perry 2013).

A function between environmental exposure to organophosphates and adverse reproductive outcomes. It has been observed that malathion causes toxicity in testis tissue with elevating reactive oxygen species (ROS) levels (Taherdehi et al. 2019). In experimental animal models, decreased sperm concentration and decreased sex hormones levels have been reported following repeated exposure to CPF. In addition, developmental disorders in spermatogenesis and damage in tubule differentiation were also detected (Babazadeh and Najafi 2017). Another main toxicity for CPF is related to acetylcholinesterase (AChE). It has been suggested that tebuconazole, a fungicide, affects spermatogenesis, induces reproductive toxicity and affects semen quality negatively (Joshi et al. 2016).

LUP exhibits a variety of the therapeutic function (Pereira Beserra et al. 2018). It is known that LUP stimulates the proliferation of skin cells, the migration of the cells, and restructuring/healing of damaged skin (Malinowska et al. 2019). LUP has been found to have cell cycle arresting and apoptosis-inducing role in cancer (Krishnendu et al. 2019).

The present study’s purpose is to the ameliorating effects of LUP (known for its antioxidant properties) against testicular toxicity caused by pesticides by histological and biochemical methods.

## Materials and methods

Our study was approved by the Ethics Committee of the Animal Experiments Local Ethics Committee of Bezmialem Vakıf University (decision no. 72-the meeting date 28.07.2021). In the study, 14-week-old Wistar albino adult male rats were obtained from Istanbul University Experimental Medicine Research Institute. The experimental process was carried out at Bezmialem Vakıf University Experimental Application and Research Center.

### Pharmacological agents and experimental design

The control group (n:7, each group) rats, constituting the first group, were given physiological saline (PS) by oral gavage and were kept alive against ageing for an equal period time. The rats in the second group were given corn oil, which was used as a solvent in the preparation of the pesticide agents to be given to all other experimental groups, by oral gavage. MAL group MAL 50 mg/kg, CPF group CPF 10 mg/kg, TEB group TEB 50 mg/kg (first 4 days)-25 mg/kg (last 4 days), LUP group 20 mg/kg LUP by oral gavage. MAL + LUP group rats received LUP 20 mg/kg + MAL 50 mg/kg, CPF + LUP group received LUP 20 mg/kg + CPF 10 mg/kg, TEB + LUP group received LUP 20 mg/kg + TEB 50 mg/kg (first 4 days)- 25 mg/kg (last 4 days) was given simultaneously by oral gavage. Before oral gavage, rats were weighed, and each chemical was administered according to body weight 8 times over 10 days. At the end of the experiment, feeding was discontinued 24 h before dissection, and the rats were only allowed to drink water. Anesthesia was performed by intramuscular injection of 50 mg/kg ketamine hydrochloride (Ketalar^®^, Eczacıbaşı) and 10 mg/kg Xylazine HCl (Alfazyne^®^, The Netherlands). The anesthetics were assessed as a surgical anaesthetic in the rat. The weighted experimental rats were dissected, the collected testicles were weighed while averages were calculated. Portions of the testicular tissues were placed in Bouin’s fixative for histological methods. They were stored at − 86 °C until required for biochemistry analyses. Testicular tissue index was created this procedure.

Testes tissue index = Weight of the testes tissue (g)/weight of the animal (g) × 100 (Mohammadi-Sardoo et al. 2018; Sretenovic et al. 2021).

### Histological preparation

The samples were prepared for staining procedure. Sections (4 μm) were stained using hematoxylin and eosin (HE). The samples were analyzed Nicon Eclipse E200 light microscope digital analysis systems. Histopathological scoring was actualized according to Hess’s criteria. Tubules were categorised as normal, regressive, degenerative or atrophic (Hess et al. 1988).

### Sperm morphological evaluation

Left caudal epididymis of all groups were dissected. Smear samples were fixed and dehydrated with 96% ethanol and stained with DiffQuick kit (Medion Diagnostics, Grafelfing, Germany) for the morphological evaluation. One hundred spermatozoa were evaluated for head, neck and tail morphology of the spermatozoa under 100x immersion oil objective of the photomicroscope.

### Biochemical evaluations

Testis tissues were homogenised in cold saline (0.9% NaCl) so as to obtain 10% (w/v) homogenates. This was followed by at 10,000 x g for 10 min and + 4 ºC. After the centrifugation process, the supernatants were collected for biochemical analysis. In the supernatants, reduced glutathione (GSH) (Beutler 1975) lipid peroxidation (LPO) (Ledwozyw et al. 1986) and advanced oxidised protein products (AOPP) (Witko-Sarsat et al. 1996) levels, catalase (CAT) (Aebi 1984), superoxide dismutase (SOD) (Mylroie et al. 1986), glutathione peroxidase (GPx) (Wendel 1981)and glutathione reductase (GR) (Beutler 1971) activities, total antioxidant capacity (TAC) (Erel 2004), total oxidant status (TOS) (Erel 2005), reactive oxygen species (ROS) (Zhang et al. 2018) and oxidative stress index (Erel 2005; Zhang et al. 2018) levels, deoxyribo nucleic acid (DNA) (Burton 1956) and nitric oxide (NO) (Miranda et al. 2001) levels, xanthine oxidase (XO) (Corte 1968) activity, alkaline and acid phosphatase (ALP and ACP) (Walter and Schutt 1974) and glucose-6-phosphate dehydrogenase (G6PD)(Beutler 1984) activities were determined. Total protein levels of the testis supernatants were evaluated according to method (Lowry et al. 1951).

### Statistical analyses

The results were evaluated using GraphPad Prism 6.0 (GraphPad Software, San Diego, California, USA). Unpaired t-test and analysis of variance (ANOVA) followed by Tukey’s multiple comparison tests.

If normality is confirmed, apply ANOVA. We checked this point.

Values of *P* < 0.05 were considered statistically significant. Statistical analysis of results was performed with Principal Component Analysis (PCA) origin program.

## Results

### Rat weights

Rat weights were measured on a precision scale. The rat weight did not show any significant change; but a reduction was seen in weights of pesticide induced groups (Table 1).

**Table 1:** Rat weight of experimental animals

Groups	Rat weights of the animals at the beginning of the experiment (g)	Rat weights of the animals at the end of the experiment (g)
Control	310.71 ± 22.47	310.42 ± 24.20
Corn oil	343.42 ± 18.51	339.57 ± 15.32
MAL	302.28 ± 14.82	300 ± 14.91
CPF	313.42 ± 12.27	290.28 ± 13.42
TEB	303.14 ± 12.75	299.42 ± 16.59
LUP	292 ± 19.52	281.71 ± 15.89
MAL + LUP	293 ± 13.39	283.28 ± 12.71
CPF+LUP	304.28 ± 14.23	287.14 ± 13.94
TEB + LUP	295.42 ± 27.41	290 ± 28.23
SD	15.72	18.06

Mean ± SD SD: Standart deviation *p* > 0.05, the results are not significant

There were a significant decrease in testes weight (*P* < 0.05) in all pesticides induced groups. A decrease in testes weight was in the control group. As a result of the administration of antioxidants to the pesticide groups, the weight difference of testis between MAL and MAL + LUP groups; between CPF and CPF + LUP group; and between TEB and TEB + LUP group was statistically increase (*P* < 0.05) (Table 2).

**Table 2:** The weight of testes of experimental animals at the end of the experiment

Groups	Testicular weight of the animals at the end of the experiment (g)*
Control	3.4 ± 0.23^b^
Corn oil	3.75 ± 0.40
MAL	2.72 ± 0.67 ^a^
CPF	2.68 ± 0.80 ^a^
TEB	3.74 ± 0.32 ^a^
LUP	3.2 ± 0.41
MAL + LUP	3.14 ± 0.34^c^
CPF + LUP	3.87 ± 0.37^d^
TEB + LUP	3.9 ± 0.66^e^

^a^*P* < 0.05 versus control group, ^b^*P* < 0.05 versus corn oil group, ^c^*P* < 0.05 versus MAL group, ^d^*P* < 0.05 versus CPF group, ^e^*P* < 0.05 versus TEB group

### Testes tissue index

The administration of antioxidants to pesticide groups resulted in an increase in testicular tissue index when comparison was done between MAL and MAL + LUP group; CPF and CPF + LUP group; and the TEB and TEB + LUP group (Table 3).

Testes tissue index = Weight of the testes tissue (g)/weight of the animal (g) × 100 (Mohammadi-Sardoo et al. 2018; Sretenovic et al. 2021). It is only a ratio for compare the groups (not statiscally result).

**Table 3:** Testes tissue index analysis between groups of experimental animals

Groups	Testes tissue index
Control	1.0
Corn oil	1.10
MAL	0.9
CPF	0.9
TEB	1.2
LUP	1.13
MAL + LUP	1.1
CPF + LUP	1.3
TEB + LUP	1.3

### Histological results

**Fig. 1:**
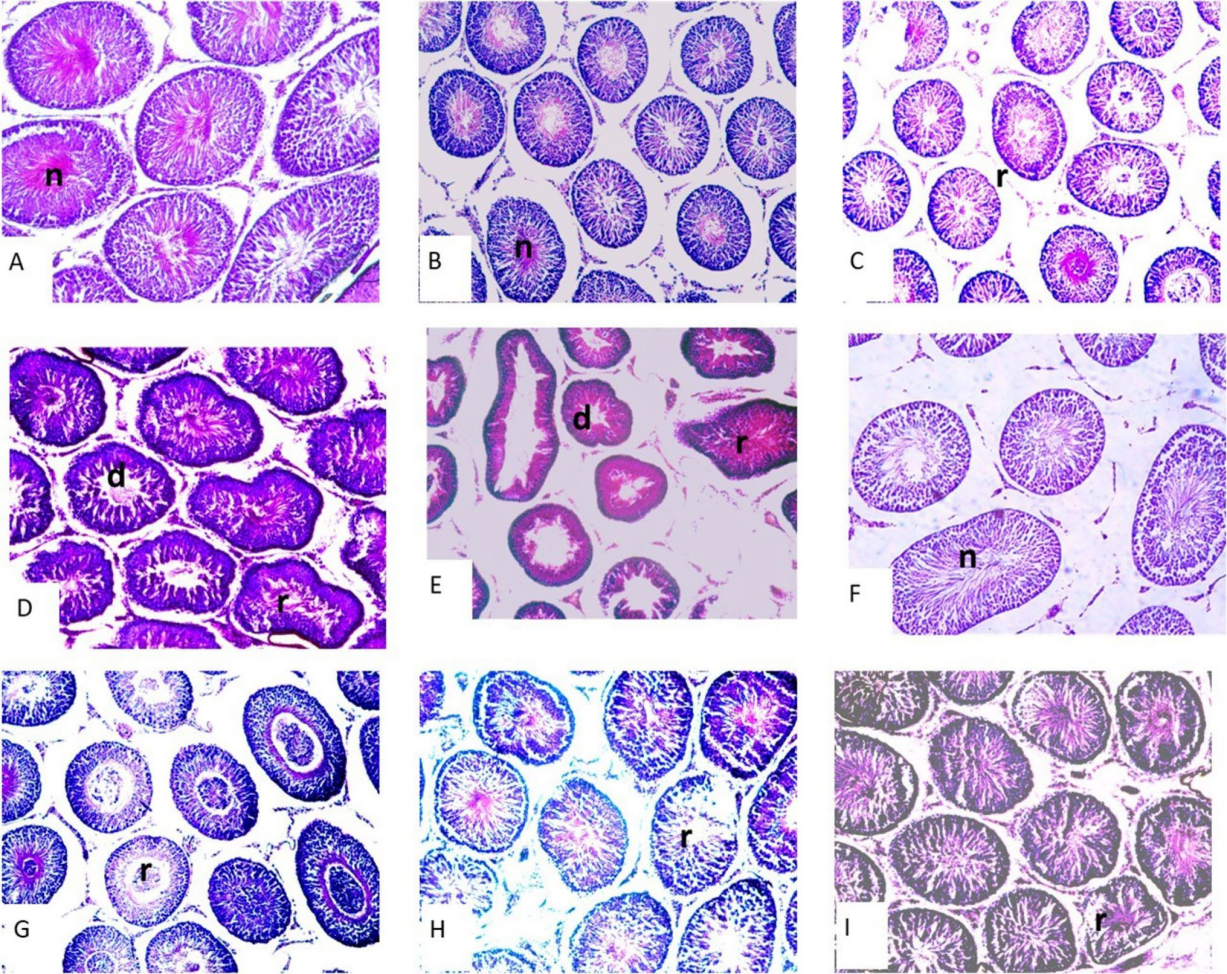
Photographs of HE stained testicular cross sections: **A** Control, **B** Corn oil **C** MAL, **D** CPF, **E** TEB, **F** LUP, **G** MAL + LUP, (**H** CPF + LUP, **I** TEB + LUP group, normal seminiferous tubules (n), regressive seminiferous tubules (r), dejenerative seminiferous tubules (d), bar: 100 μm

When testicle sections stained with HE (Fig. 1), the control and corn oil group depicts a normal seminiferous tubule organsiation. The MAL, CPF and TEB groups (pesticide induced groups) showed progressively tubular degeneration. Many seminiferous tubules showed normal structure, and spermatogenic cycle cells were also normal with lupeol treatment.

### Testicular damage score

**Fig. 2:**
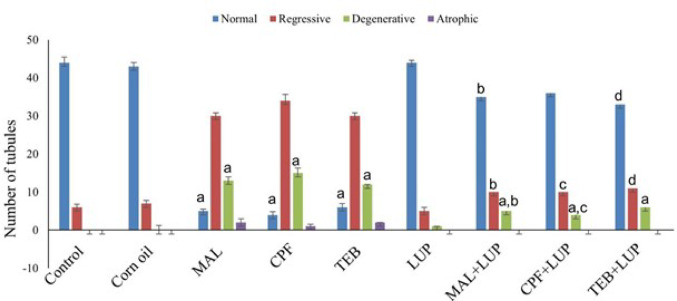
The histopathological damage score of testicular tissue, with the number of normal, regressive, degenerative and atrophic seminiferous tubules in groups. a: *P* < 0.01 versus control group, b: *P* < 0.001 versus MAL group, c: *P* < 0.05 versus CPF group, d: *P* < 0.001 versus TEB group

The seminiferous tubule structure of testes in the control and corn oil groups were normal, but the normal tubule score decreased in all other groups. The normal tubule decrease was higher (*P* < 0.001) in the MAL, CPF and TEB groups. Normal tubule number was seen (*P* < 0.01) in LUP treated group. Degenerative tubule numbers in the LUP treated group were similar to that of the control group. Following LUP application, histologic damages in the testes of pesticide induced experimental animals were reduced, and an increased number of normal seminiferous tubules (Fig. 2).

### Sperm smear results

**Fig. 3:**
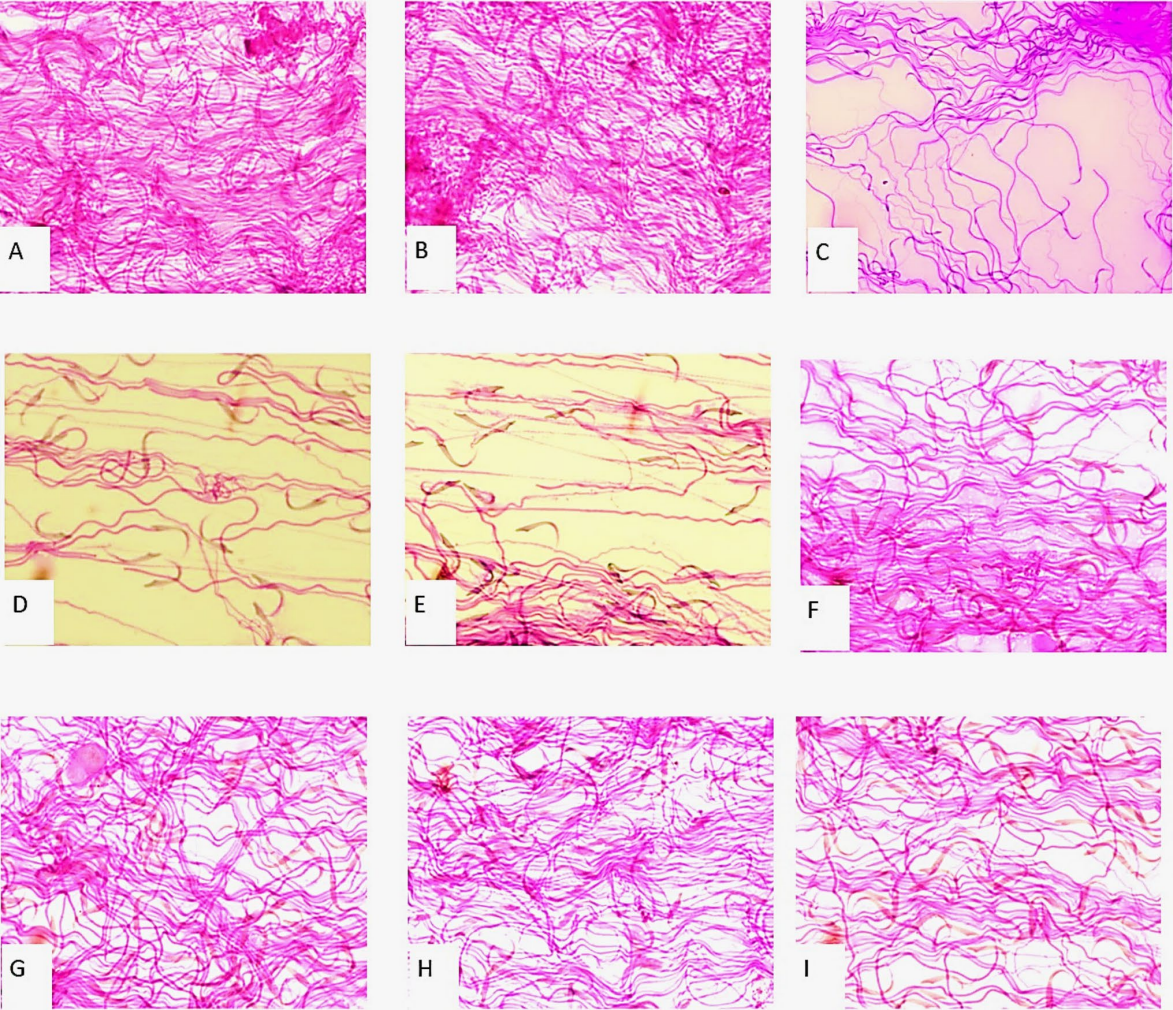
Photographs of sperm smear sections: **A** Control, **B** Corn oil **C** MAL, **D** CPF, **E** TEB, **F** LUP, **G** MAL + LUP, **H** CPF + LUP, **I** TEB + LUP group, bar: 100 μm

The spermatozoa with normal morphology and abnormal spermatozoa presenting head, midpiece and tail defects were observed in all groups. Head, midpiece, and tail defects were significantly increased in the pesticide induced group compared to control group. Some sperm morphological defects were significantly decreased in the LUP treated groups compared to the pesticide induced groups (Fig. 3).

### Biochemical results

**Fig. 4:**
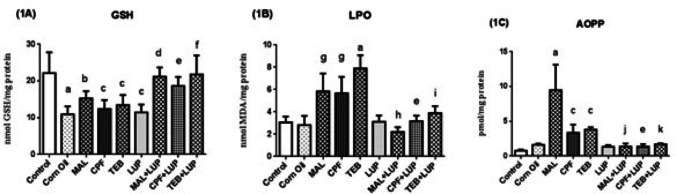
The testis GSH (**1A**), LPO (**1B**) and AOPP (**iC**) levels of control and experimental groups. Data are expressed as “mean ± standard deviation”. ^a^*P* < 0.0001 vs. control group; ^b^*P* < 0.05 versus control group; ^c^*P* < 0.001 versus control group; ^d^*P* < 0.05 versus MAL group, ^e^*P* < 0.05 versus CPF group; ^f^*P* < 0.001 versus TEB group; ^g^*P* < 0.01 versus control group; ^h^*P* < 0.01 versus MAL group; ^i^*P* < 0.0001 versus TEB group; ^j^*P* < 0.0001 versus MAL group; ^k^*P* < 0.05 versus TEB group

The testis GSH (1A), LPO (1B) and AOPP (1C) levels are indiaceted in Fig. 4. Testis GSH levels were found to be significantly decreased after corn oil and LUP treatment in the control group (^a^*P* < 0.0001, ^c^*P* < 0.001). MAL, CPF and TEB caused a decrease in GSH levels, respectively (^b^*P* < 0.05; ^c^*P* < 0.001). In contrast, LUP administration ameliorated testis GSH levels of MAL, CPF and TEB, respectively (^d^*P* < 0.05, ^e^*P* < 0.05, ^f^*P* < 0.001). LPO and AOPP levels were significantly increased after MAL, CPF and TEB administration in the control group, respectively (^g^*P* < 0.01, ^a^*P* < 0.0001, ^c^*P* < 0.001). LUP reversed these levels in a significant manner in the MAL, CPF and TEB group, respectively (^h^*P* < 0.01, ^e^*P* < 0.05, ^i^*P* < 0.0001, ^j^*P* < 0.0001, ^e^*P* < 0.05, ^k^*P* < 0.05).

**Fig. 5:**
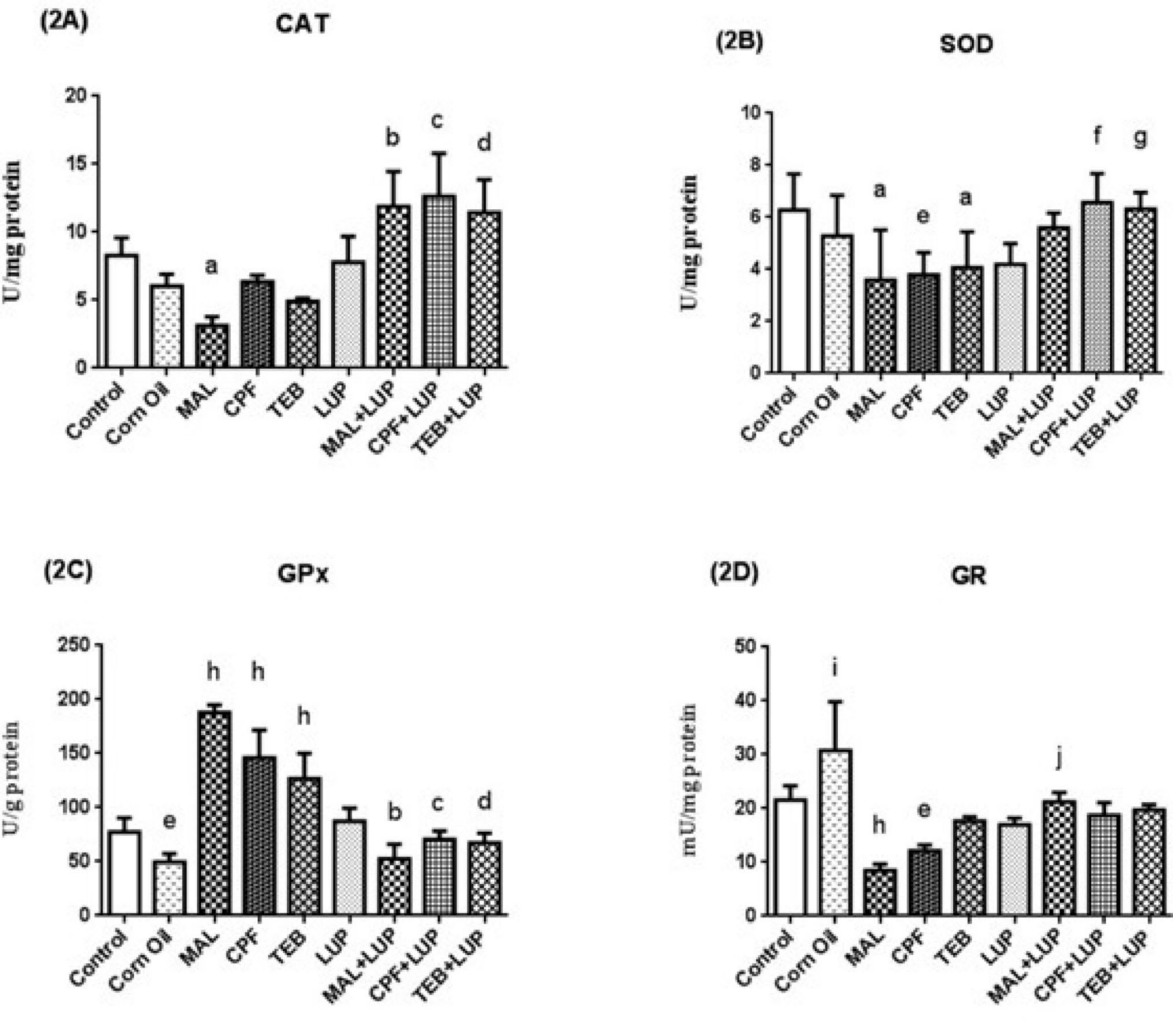
The testis CAT (**2A**), SOD (**2B**), GPx (**2C**) and GR (**2D**) activities of control and experimental groups. Data are expressed as “mean ± standard deviation”. ^a^*P* < 0.05 versus control group; ^b^*P* < 0.0001 versus MAL group; ^c^*P* < 0.0001 versus CPF group; ^d^*P* < 0.0001 versus TEB group; ^e^*P* < 0.01 versus control group; ^f^*P* < 0.001 versus CPF group; ^g^*P* < 0.05 versus TEB group; ^h^*P* < 0.0001 versus control group; ^i^*P* < 0.001 versus control group; ^j^*P* < 0.001 versus MAL group

The testis CAT (2A), SOD (2B), GPx (2C) and GR (2D) activities are presented in Fig. 5. Corn oil application decreased GPx and increased GR activities in the control group (^e^*P* < 0.01, ^i^*P* < 0.001, respectively). MAL significantly decreased CAT activities (^a^*P* < 0.05), while the diminishment effects of CPF and TEB on CAT were statistically insignificant as compared to control groups.Pesticides decreased SOD activities and increased GPx activities of the control group (^a^*P* < 0.05, ^e^*P* < 0.01 for SOD activities; ^h^*P* < 0.0001 for GPx activities, respectively). MAL and CPF significantly decreased GR activities (^h^*P* < 0.0001, ^e^*P* < 0.01), while TEB administration effect was statistically insignificant. Administration of LUP reversed CAT and GPx activities in MAL, CPF and TEB groups (^b^*P* < 0.0001, ^c^*P* < 0.0001, ^d^*P* < 0.0001, respectively), SOD activities in CPF and TEB groups (^f^*P* < 0.001, ^g^*P* < 0.05), and GR activity in MAL group (^j^*P* < 0.001) were also reversed bu LUP in a significant manner.

**Fig. 6:**
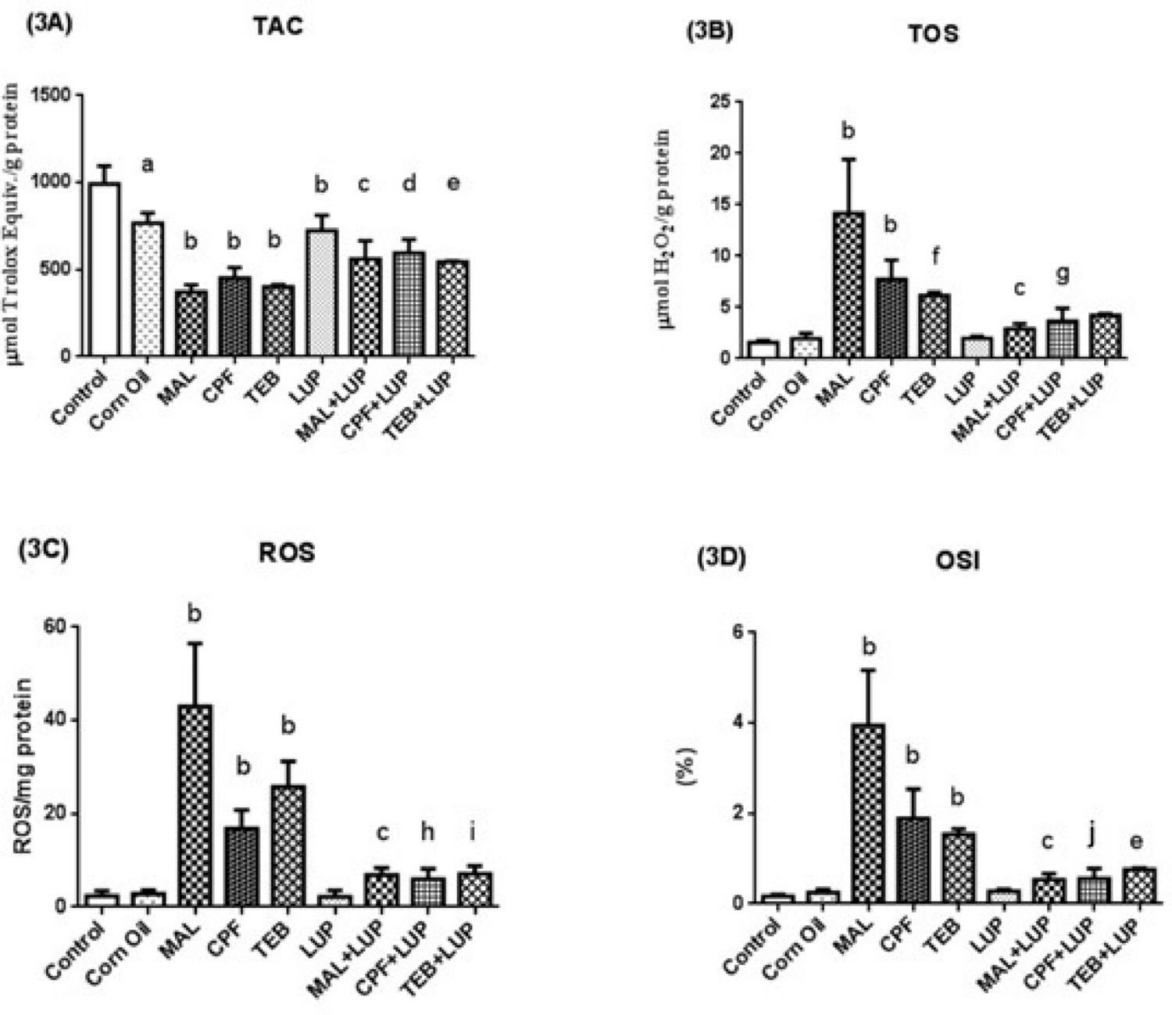
The testis TAC (**3A**), TOS (**3B**), ROS (**3C**) and OSI (**3D**) levels of control and experimental groups. Data are expressed as “mean ± standard deviation”. ^a^*P* < 0.001 versus control group; ^b^*P* < 0.0001 versus control group; ^c^*P* < 0.0001 versus MAL group; ^d^*P* < 0.05 vesus CPF group; ^e^*P* < 0.05 versus TEB group; ^f^*P* < 0.01 versus control group; ^g^*P* < 0.001 versus CPF group; ^h^*P* < 0.01 versus CPF group; ^i^*P* < 0.0001 versus TEB group; ^j^*P* < 0.0001 versus CPF group

The testis TAC (3A), TOS (3B), ROS (3C) and OSI (3D) activities are given in Fig. 6. Corn oil and LUP significantly decreased TAC levels of the control group (^a^*P* < 0.001, ^b^*P* < 0.0001, respectively). MAL, CPF and TEB administration diminished TAC levels of control groups in a significant manner (^b^*P* < 0.0001, respectively). LUP on the other hand significantly ameliorated TAC levels in MAL, CPF and TEB group (^c^*P* < 0.0001, ^d^*P* < 0.05, ^e^*P* < 0.05). TOS, ROS and OSI levels were significantly increased after MAL, CPF and TEB administration to the control group (^b^*P* < 0.0001, ^f^*P* < 0.01, respectively). LUP decreased TOS levels in MAL + LUP and CPF + LUP group (^c^*P* < 0.0001, ^g^*P* < 0.001, respectively), as well as ROS and OSI levels in MAL + LUP, CPF + LUP and TEB + LUP groups in a significant manner (^c^*P* < 0.0001, ^h^*P* < 0.01, ^i^*P* < 0.0001, ^j^*P* < 0.0001, ^e^*P* < 0.05, respectively).

**Fig. 7:**
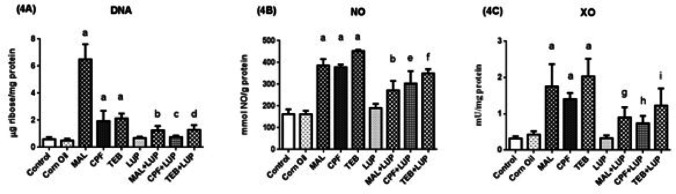
The testis DNA (**4A**) and NO (**4B**) levels, XO (**4C**) activities of control and experimental groups. Data are expressed as “mean ± standard deviation”. ^a^*P* < 0.0001 versus control group, ^b^*P* < 0.0001 versus MAL group; ^c^*P* < 0.0001 versus CPF group; ^d^*P* < 0.05 versus TEB group, ^e^*P* < 0.01 versus CPF group; ^f^*P* < 0.0001 versus TEB group; ^g^*P* < 0.001 versus MAL group; ^h^*P* < 0.05 versus CPF group; ^i^*P* < 0.01 versus TEB group

The testis DNA (4A) level, NO (4B) levels, and XO (4C) activities are given in Fig. 7. According to the results, statistically significant elevation of DNA and NO levels, coupled with increased XO activities were observed in the pesticides groups when compared to the control group (^a^*P* < 0.0001). Administration of LUP to the MAL, CPF and TEB groups reversed these defects in a significant manner (^b^*P* < 0.0001, ^c^*P* < 0.0001, ^d^*P* < 0.05, ^e^*P* < 0.01, ^f^*P* < 0.0001, ^g^*P* < 0.001, ^h^*P* < 0.05, ^i^*P* < 0.01, respectively).

**Fig. 8:**
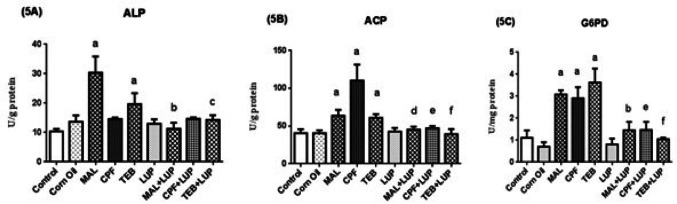
The testis ALP (**5A**), ACP (**5B**) and G6PD (5C) activities of control and experimental group. Data are expressed as “mean ± standard deviation”. ^a^*P* < 0.0001 versus control group; ^b^*P* < 0.0001 versus MAL group; ^c^*P* < 0.01 versus TEB group; ^d^*P* < 0.001 versus MAL group; ^e^*P* < 0.0001 versus CPF group; ^f^*P* < 0.0001 versus TEB group

The ALP (5A), ACP (5B) and G6PD (5C) activities of testis are given in Fig. 8. ALP activities were significantly increased (^a^*P* < 0.0001), while the ALP elevation in CPF given group was statistically insignificant. In comparison to control groups, ACP and G6PD activities were significantly increased after pesticides induced (^a^*P* < 0.0001). LUP significantly decreased ALP activities of MAL and TEB groups, respectively (^b^*P* < 0.0001, ^c^*P* < 0.01). The Administration of LUP did not confer ameliorative effect to the altered ALP activities of CPF group. In MAL + LUP, CPF + LUP and TEB + LUP groups, ACP and G6PD activities were decreased as compared to MAL, CPF and TEB groups, respectively (^d^*P* < 0.001, ^e^*P* < 0.0001, ^f^*P* < 0.0001, ^b^*P* < 0.0001, ^e^*P* < 0.0001).

### PCA analysis result

**Fig. 9:**
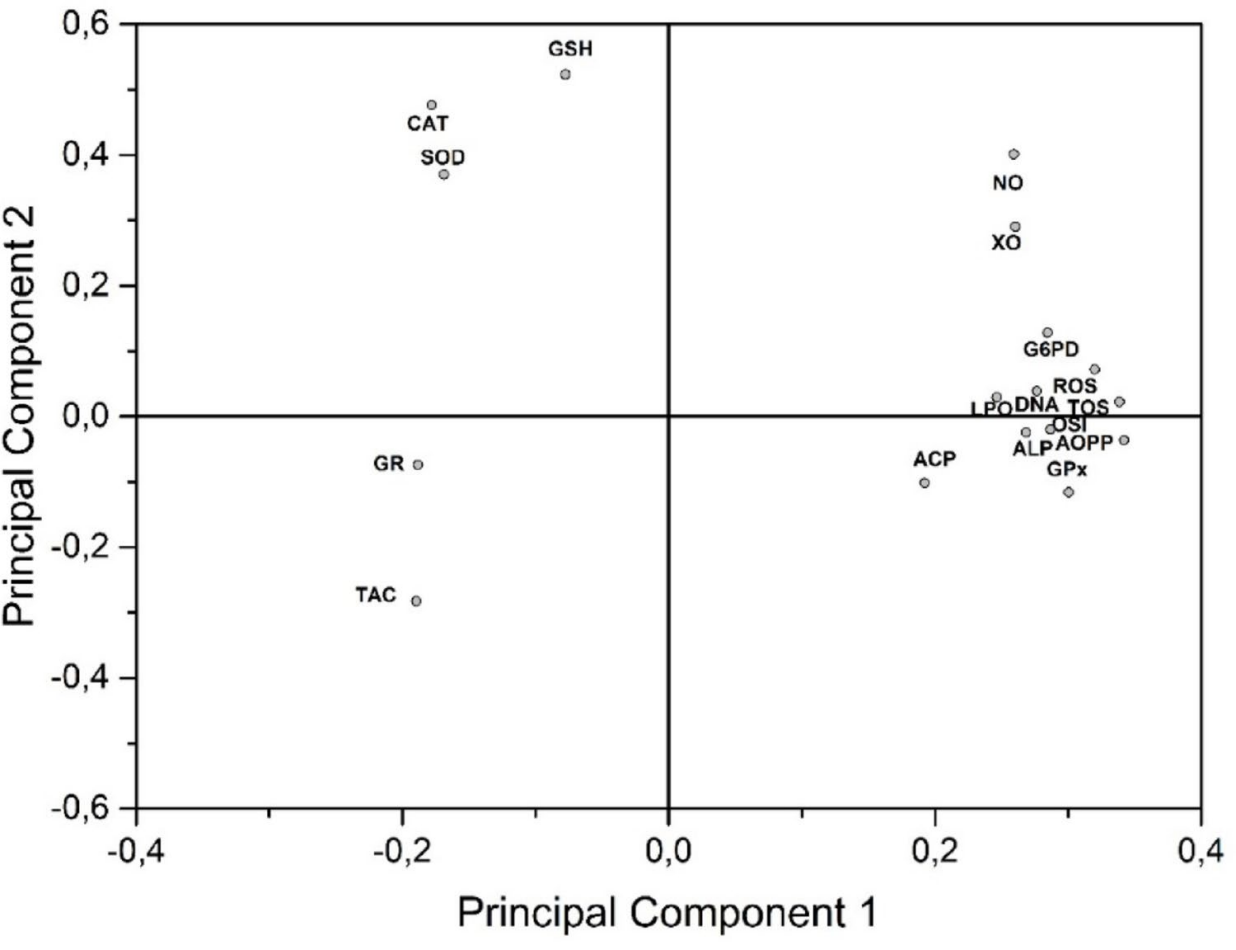
Principal component analysis plot analysis showing. All biochemical results indicator plotted as a function of two first components (PC1: 61.39%, PC2: 10.11%, total PC: 71.50%)

**Fig. 10:**
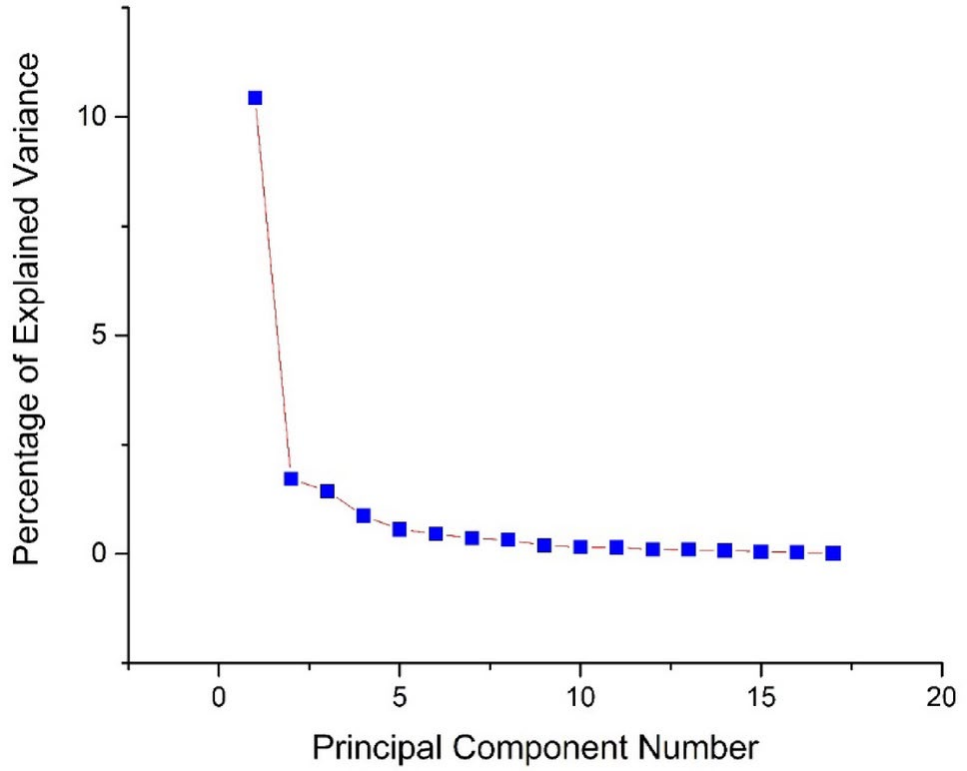
Eigenvalues analysis of all biochemical results are shown

The correlation between biochemical endpoints has been determined with PCA in order to show lupeol efficacity. PCA has shown that PC1 explained 61.39% whereas PC2 explained that 10.11% of the total variation. NO, XO, G6PD, ROS, TOS, LPO, DNA, ACP, ALP, GPx, and AOPP biomarkers have displayed a positive correlation with oxidative stress markers whereas TAC, GR, CAT, SOD, and GSH have shown a negative correlation with them (Figs. 9 and 10).

## Discussion

Pesticides can directly damage spermatozoa (Gangemi et al. 2016). These examinations determined that the number of spermatozoa decreased and the incidence of regressive and degenerate spermatozoa increased in pesticide induced groups. In addition, pesticides have been shown to impair testes and sperm morphology. LUP treatment improves pesticide induced sperm parameters and testicular damage.

Pesticides have a relatively short biological half-life (Burns et al. 2013). The pesticides mostly exhibit toxicity by inhibiting AChE enzyme, which breaks down Ach (Mladenović et al. 2018; Shaffo et al. 2018).

Pesticide cause morphological alterations and cell death in mitochondria even at low concentrations (Chen et al. 2017). CPF causes histological damage to testicular tissue, sperm motility and immature sperm. It is effective in reducing sperm DNA, sperm count, motility and vitality (Babazadeh and Najafi 2017). Endosulfan that spermatogenesis was affected, epididymal sperm count and viability decreased, epididymal ROS increased, and sperm chromatin integrity was also affected (Sebastian and Raghavan 2015).

There is sufficient evidence that pesticide exposure is associated with decreased sperm motility, concentration, and DNA integrity, and spermatotoxicity. Various agricultural policies should be implemented to reduce exposure by reducing pesticide use. Agricultural pesticides also act as androgen antagonists. They are direct effects on sperm cells through reactive oxygen species and free radicals that damage cell membranes, organelles, and DNA. Spermatozoa are susceptible to oxidative damage from ROS because sperm cell membranes are largely composed of unsaturated fatty acids that are subject to oxidation, and the sperm cytoplasm has low concentrations of enzymes that neutralize ROS. Damage to sperm DNA occurs with decreased sperm parameters and affects male fertility (Knapke et al. 2022).

It was concluded that CPF causes severe testicular damage, it is emphasised that pesticide use should be limited (Joshi et al. 2007). When testicular tissue damage caused by a fungicide named Mancozeb was examined in an experimental model, testicular dysfunction was observed to have occurred through oxidative stress and apoptosis. When the antioxidant effects of N-acetylcysteine, a modified amino acid, were examined, it was found that it reversed the damage (Mohammadi-Sardoo et al. 2018). Cypermethrin use showed a significant increase testicular damage score. Treatments with resveratrol reverses damage parameters of the pesticide group (Sharma et al. 2014).

When the effects of LUP on tumour and endothelial cells were examined in an experimental model, it was found to have suppressed tumour angiogenesis and inhibited carcinoma growth through tumour necrosis factor (Kangsamaksin et al. 2017; Saleem et al. 2009). LUP was found to inhibit the elevation of digestive enzymes. Kim et al. suggested that LUP exhibited protective effects on cerulein-induced acute pancreatitis (Kim et al. 2015).

LUP was shown to provide significant protection and diminishing acetaminophen-induced toxicity (Kumari and Kakkar 2012).

GSH, despite being a small tripeptide, is one of the vital antioxidant molecules which host many regulations on antioxidant systems, and lead to unique protection for many tissues. Considering its protective effect on testis tissue in particular, the protection by GSH due to its positive effects on sperm quality and testis morphology as proven in a diabetes study cannot be ignored (Abdullah et al. 2021). MDA is an important oxidised compound (Ghosh et al. 2002). AOPP is another peroxidation product which indicates cellular protein damage arising from excess ROS. These parameters are strongly affected by various pesticides exposures which have been reported by many researchers and the results of the present study (Oksay et al. 2013; Mahajan et al. 2018; Zhang et al. 2020a). Administration of LUP reversed these defects in pesticide given groups. The molecules/substances which have pentacyclic triterpene structure like LUP and lantadene that have antioxidant activity (Nagaraj et al. 2000; Grace-Lynn et al. 2012). In the light of this information, it may be assumed that LUP helped the amelioration on GSH, LPO and AOPP levels by its proven antioxidant effect.

Oxidative stress which causes cell death and other complex metabolic diseases like diabetes mellitus and cancer (Halliwell 1994). Pesticides like fungicides and other substances which have organophosphate structure are known producers, elevators, and accumulators of free radicals (Srivastava et al. 2016; Zhang et al. 2020b; Freitas et al. 2021). These effects may be promoted by converting the P = S (thion) bond of organophosphates to P = O (oxon) thereby further inducing oxidative stress (accepted for MAL and CPF), and triazole ring-hydroxy and Cl^−^ part of TEB (Vidyasagar et al. 2004; Fortunato et al. 2006; Raina et al. 2015; Želonková et al. 2019). CAT, SOD, GPx, GR and GST activities, in addition to TAS, TOS, ROS and OSI levels were determined for evaluating ROS triggering levels of MAL, CPF and TEB. Testis CAT, SOD and GR activities and TAS levels were decreased in pesticides given groups, while GPx activity, TOS, ROS and OSI levels were elevated in the current study. To explain these results, the diminished GSH and increased LPO may have arised due to the excess formation of hydrogen peroxide and superoxide anion radicals. These altered GSH-LPO levels indicate increased GPx activity due to increased H_2_O_2_ which may have been scavenged by this enzyme and this approach may explain the diminished CAT activities. Our results for antioxidant enzymes support the explanation of TAS, TOS, ROS and OSI level alterations. LUP has been shown to have the ability to scavenge superoxide anions rapidly by its methyl part at C17 position in experimental hypercholesterolemia (Sudhahar et al. 2007). In addition to that, they explained the importance of aromatic ring proximity and hydroxyl groups having great potential for pentacyclic triterpenes (Hagerman et al. 1998). It put forward that these parts can scavenge most of the radicals. In the light of this information, we may hypothesise that LUP showed an excellent antioxidant property as ameliorating antioxidant system (Grace-Lynn et al. 2012).

DNA damage were reported in a study involving Greenhouse workers who were exposed to pesticides (Çayır et al. 2018). In the present study, increased DNA levels with elevated XO activities (which indicate the increased purine catabolism) in testis samples of pesticide given groups were observed. These findings indicate both the direct effect of these pesticides on DNA structure or increased free radical levels, which can also explain the impaired purine structure by elevating XO. LUP inhibits lyase activity of DNA polymerase beta and protect cancer tissues against the damaged-DNA agents (Sobol et al. 2000; Chaturvedula et al. 2004). LUP may have reversed these parameters by protecting testis tissue via its antioxidant activity.

NO is vital power for reproductive system (Tejero et al. 2019). However, excess NO is highly toxic for sperms, and is associated with elevator effect on LPO via peroxynitrite formation, in turn peroxidative damage and tyrosine nitrosylation (Rosselli et al. 1998; Darley-Usmar et al. 2000). MAL, TEB and CPF administration lead to increase NO formation in testis tissue. The administration of LUP reversed NO level in the testis of pesticide given experimental groups’ probably by its antioxidant property.

ALP plays a role in breaking down phosphate esters. Their elevated activities have been reported during degeneration and cellular disintegration, dilated appearance in blood capillaries in Leydig and spermatogenic cells that occurred due to pesticide intoxication (Mahgoub and El-Medany et al. 2001). In the present study, elevated activities of these enzymes were observed. These elevations indicate that pesticides do harm the testis tissue. The administration of LUP containing plants or the direct administration of LUP have been reported as showing an ameliorative effect on these enzymes in different experimental models (Preetha et al. 2006; Khalaf-Allah et al. 2016). In this study, LUP showed its protection by decreasing the activity of these enzymes in testis tissue.

G6PD is a key metabolic enzyme related to pentose phosphate pathway, as supplementer for reducing equivalents for steroid hydroxylation (Abd El Tawab et al. 2014). The main need for this enzyme activity is dependent on NADPH production which occurs via PPP. In this study, elevated G6PD in MAL, TEB, and CPF given groups was observed. This elevation can be associated with increased requirement of NADPH by altered antioxidant status due to pesticides. LUP administration reversed this defect probably by its effect on the antioxidant system.

## Conclusion

In the present study, damages were found in the testes of rats treated with CPF, MAL and TEB in line with the literature. In the experimental model, it was observed that there was a decrease in the weight of animals exposed to pesticides at the end of the experiment period. A statistically significant increase in testicular weights was observed upon treatment of the pesticides groups with LUP when compared to the pesticides groups. While a normal testicular histology was observed in the control and corn oil groups, the number of regressive and degenerative tubules increased in the pesticide groups; It was observed that the basement membrane structure was disrupted, and the number of spermatozoa decreased. Sperm smear findings also support testicular histology; sperm anomalies were observed at a higher rate in the pesticide groups, with disorders in the head, neck and tail. LUP administration has been observed to reverse all these findings. It can be suggested that LUP application has a protective and healing effect on the oxidative damage that may occur by eliminating the ROS evolved by pesticides in the testis.

## Data Availability

No datasets were generated or analysed during the current study.
